# Shapes of ideal stalagmites

**DOI:** 10.1073/pnas.2513263122

**Published:** 2025-10-16

**Authors:** Piotr Szymczak, Anthony J. C. Ladd, Matej Lipar, Dean Pekarovič

**Affiliations:** ^a^Institute of Theoretical Physics, Faculty of Physics, University of Warsaw, Warsaw 02-093, Poland; ^b^Department of Chemical Engineering, University of Florida, Gainesville, FL 32611; ^c^Anton Melik Geographical Institute, Research Centre of the Slovenian Academy of Sciences and Arts, Ljubljana 1000, Slovenia; ^d^Clinical Radiology Institute, University Medical Centre Ljubljana, Ljubljana 1000, Slovenia

**Keywords:** stalagmite formation, speleothems, invariant growth, nonlinear physics

## Abstract

Stalagmites are isolated columns of calcium carbonate growing on a cave floor; their growth is driven by the constant dripping of supersaturated solutions from the roof of the cave. In this paper, we derive a closed-form expression for the shape of a steadily growing stalagmite. Our analysis gives rise to three distinct shapes, all of them observable in nature, with the shape characterized by a single dimensionless parameter. Transitions between different shapes occur at a specific value of this parameter, with additional selection rules determining the shape and size of stalagmites evolving under specific cave conditions. Our theory shows that the stalagmite shape influences the ^13^C isotope shifts, which are an important source of paleoclimatic information.

## Significance

Stalagmites come in a variety of shapes, with sizes ranging from centimeters to meters. Despite their intriguing regularity and their importance for paleoclimate studies, a theoretical description of their form has been lacking. We have developed an analytic theory that provides an explicit formula for the shapes of steadily growing stalagmites, and characterizes the variety of observed shapes in terms of a single parameter. Our results provide a unified perspective on different numerical approaches to stalagmite growth, and make connections to theories of stalactite growth as well. The results can also be used to help infer paleoclimatic information derived from isotope measurements.

**Fig. 1.**
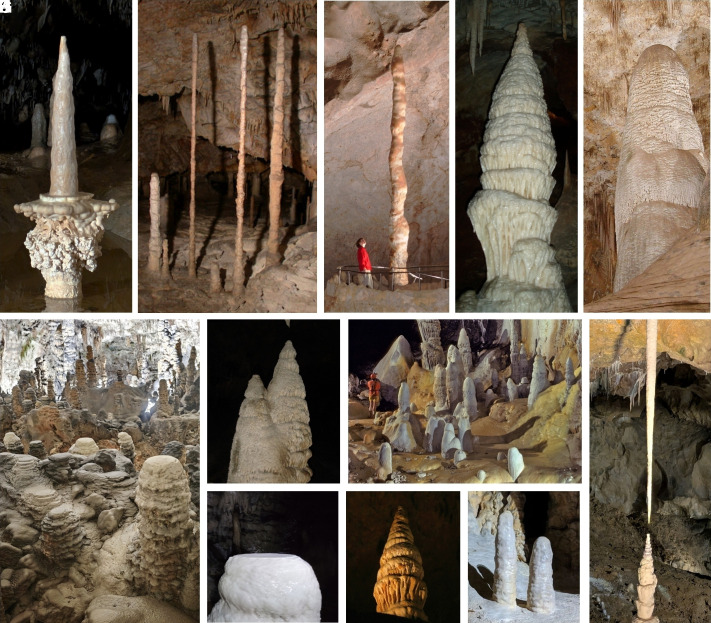
(*A*) “Candlestick,” conical stalagmite in Sloupsko-šošůvské Caves, Czechia; (*B*) long columnar stalagmites in Kateřinská Cave, Czechia; (*C*) “Witch’s Finger” columnar stalagmite in Carlsbad Caverns, USA; (*D*) “Minaret” stalagmite in Jenolan Caves, Australia; (*E*) columnar stalagmite in Carlsbad Caverns, USA; (*F*) flat-top stalagmites in Postojna Cave, Slovenia; (*G*) conical stalagmites in Carlsbad Caverns, USA; (*H*) columnar stalagmites in Lechuguilla Cave, New Mexico, USA; (*I*) “Romeo and Juliet” stalactite–stalagmite pair in Punkva Caves, Czechia; (*J*) flat-top stalagmite in Postojna Cave, Slovenia; (*K*) “Wedding cake” stalagmite in Luray Caverns, Virginia, USA; (*L*) Columnar stalagmites in Yonderup Cave, Australia. Photo credits: (*A*, *I*, and *K*): Piotr Szymczak, (*B*): Jochen Duckeck (public domain), (*C* and *E*): Peter Jones, National Park Service, USA (public domain), (*D*): Bellman (public domain), (*F* and *J*): Matej Lipar, (*G*): Paul J. Morris (CC BY-SA 2.0), (*H*): Dave Bunnell/Under Earth Images (CC BY-SA 2.5), and (*L*): Andy Baker (reproduced with permission).

Stalagmites grow from cave floors by precipitation of calcium carbonate from oversaturated water dripping from the roof of the cave (1–4). Examples of their diverse morphologies are shown in Fig. 1. Their forms have long captured our imagination, with individual stalagmites of particular beauty given whimsical names that reflect their appearance; for example “Minaret” (Fig. 1*D*), “Witch’s Finger” (Fig. 1*C*), “Romeo and Juliet” (Fig. 1*I*), or “Wedding Cake” (Fig. 1*K*). Apart from their aesthetic appeal, stalagmites are a rich source of information about the paleoclimate, with successive layers of mineral deposition playing a similar role to tree rings in studies of climate history (5–7).

Stalagmite formation is driven by geochemical processes initiated when subsurface water absorbs carbon dioxide from soil respiration, increasing its acidity (6). This acidic water then dissolves limestone, releasing calcium ions into the solution. Upon entering the cave, the water encounters a lower partial pressure of carbon dioxide (*p*CO_2_) in the cave atmosphere, leading to CO_2_ degassing. This shifts the carbonate equilibrium, resulting in oversaturation with respect to calcium carbonate, which then precipitates to form speleothems such as flowstones, stalactites, and stalagmites.

Nearly sixty years ago, Franke (1) formulated a mathematical model for the growth of stalagmites. In this model, the local growth rate of a stalagmite is proportional to the oversaturation of calcium ions in the solution dripping over the surface of the stalagmite. Franke postulated that, if the physical conditions in the cave remain constant, then after a sufficiently long time, the stalagmite will assume a certain ideal shape, which in further stages of growth will only move upward without further changing its form. These conclusions were later confirmed in computer simulations (5, 8–11); however, the mathematical form of this ideal shape was not found.

As we will show, Franke’s model of stalagmite growth can be solved analytically, leading to invariant, Platonic forms of stalagmites that could be observed in an “ideal cave,” under constant physical conditions and with a constant flow of water dripping from the associated stalactite. Interestingly, it turns out that the columnar shape (Fig. 1*B*, *C*, *E*, *H*, and *L*) found numerically in refs. 8 and 11 is only one of a family of shapes and sizes, characterized by the Damköhler number Da=kA/Q; here, k is the rate constant of the precipitation reaction, A is the cross-sectional area at the base of the stalagmite, and Q is the volumetric flow rate of water dripping onto the stalagmite. These new solutions describe stalagmites with a flat top of fixed diameter (Fig. 1 *F* and *J*), as well as conical stalagmites (Fig. 1*A*, *D*, *G*, and *K*) with sharply pointed tops.

Through their gradual and persistent growth, stalagmites not only create mesmerizing subterranean landscapes but also act as chronological records of Earth’s geological and climatic past, offering insights into the history of their respective cave environments. Our work integrates previous studies of stalagmite formation (1, 5, 8–12) into a unified picture of the growth of ideal stalagmites. We indicate how these analytic solutions can be used to extract more extensive and reliable information from measurements of isotope shifts.

## 1. Mathematical Model

**Fig. 2.**
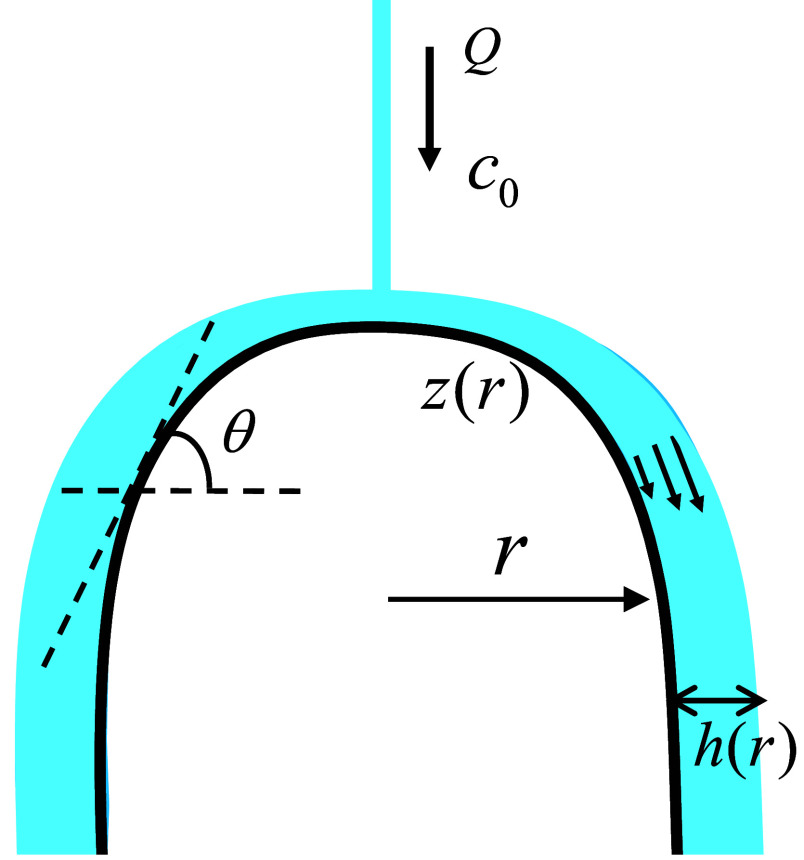
Sketch of a columnar stalagmite with a monotonic surface z(r). The local tangent to the surface defines the angle θ. The water film is indicated in blue.

We envision oversaturated water, dripping from a stalactite hanging from the cave’s ceiling, and impinging on a cylindrically symmetric stalagmite whose surface is represented by the monotonic function z(r). A sketch of the geometry is shown in Fig. 2. A stream of water, with a volumetric flow rate Q, and calcium-ion concentration $c_{0}$, drips onto the apex of the stalagmite and flows tangentially across the surface, symmetrically enveloping the entire stalagmite with a thin film of water (Fig. 2). The film thickness can be correlated with the local slope (tanθ) through the thin-film equations (13). Assuming a stress-free boundary condition at the air–water interface, the velocity field in the film is parabolic, and the film thickness h(r) follows from the volumetric flux of the water stream:

[1] $$\begin{array}{c} \frac{Q}{2\pi r}=\frac{h^{3}}{3\nu }g\sin\theta . \end{array}$$

We assume that the growth rate of a stalagmite is determined by the local oversaturation of calcium ions $c-c_{\text{sat}}$ (1, 11, 14), where c is the concentration of $Ca^{2+}$ ions within the fluid film and $c_{\text{sat}}$ is the saturation concentration. The growth of the stalagmite is then described by the evolution of the surface z(r,t):

[2] $$\begin{array}{c} \partial_{t}z=\nu_{\text{M}}\frac{J(c)}{\cos\;\theta }, \end{array}$$

where $\nu_{\text{M}}$ is the molar volume of calcium carbonate and 1/cosθ accounts for the normal growth of the surface. The rate of precipitation in karst systems is, to a good approximation, linear in the oversaturation (15), with the reaction rate

[3] $$\begin{array}{c} J\left(c\right)=k\left(c-c_{\text{sat}}\right), \end{array}$$

where $k=1.3\times 10^{-5}$ cm/s is the precipitation rate constant (11).

Since the film thickness (h) is small, diffusion is fast compared to reaction, kh/D≪1. Consequently, the concentration across the film can be considered uniform. On the other hand, tangential diffusion is usually negligible in comparison with convection, with the Péclet number Pe=Q/(2πhD)≫1. The transport equation is then simply a balance of convection and reaction,

[4] $$\begin{array}{c} \frac{1}{2\pi r}\partial_{r}\left(Qc\right)=-\frac{k(c-c_{\text{sat}})}{\cos\;\theta }, \end{array}$$

supplemented by the boundary condition $c\left(r=0\right)=c_{0}$. A detailed derivation of Eq. **4** is provided in *SI Appendix*.

The coupled equations describing the entire evolution of the stalagmite (Eqs. **2**–**4**) can only be solved numerically (11). However, it is significantly simpler to find an invariant solution that propagates upward without changing its shape, so that $z\left(r,t\right)-z_{\text{apex}}\left(t\right)$ becomes time independent. Such invariant solutions are encountered across many different pattern-forming systems. A well-known example is the Saffman–Taylor finger, which emerges as an asymptotic solution in viscous fingering (16), or the Ivantsov paraboloid (17), which is the corresponding solution in solidification. Other examples include the regular shapes of flames (18) or crystals growing in a capillary (19). In natural systems, a similar concept was used to describe karst pinnacles (20, 21), solution pipes (22), icicles (23, 24), travertine cones (25) or valley heads formed by seepage erosion (26).

Invariant growth is characterized by a constant velocity in the vertical direction, $U=\partial_{t}z\left(r,t\right)$. Eq. **2** can then be used to connect the concentration field to the shape:

[5] $$\begin{array}{c} \left(c-c_{\text{sat}}\right)=\frac{U}{k\nu_{\text{M}}}\cos\;\theta . \end{array}$$

After applying the invariance condition (Eq. **5**), the transport equation (Eq. **4**) can be integrated to obtain the concentration profile

[6] $$\begin{array}{c} c=c_{0}-\pi r^{2}\frac{U}{Q\nu_{\text{M}}}. \end{array}$$

An immediate consequence of Eq. **6** is that there is a well-defined radius R, where the oversaturation vanishes, $c\left(R\right)=c_{\text{sat}}$:

[7] $$\begin{array}{c} R=\sqrt{\frac{Q\nu_{\text{M}}}{\pi U}\left(c_{0}-c_{\text{sat}}\right)} \end{array}$$

From Eq. **5**, θ(R)=π/2, so r=R also corresponds to the vertical sides of the stalagmite. This connects the maximum radius of the stalagmite to the propagation velocity, which can also be found by balancing the incoming flux of reactant $Q(c_{0}-c_{\text{sat}})$ with the precipitation rate $\pi R^{2}U/\nu_{M}$.

The tangent angle can be obtained by substituting the concentration (Eq. **6**) into the invariance condition (Eq. **5**):

[8] $$\begin{array}{c} \cos\;\theta \left(r\right)=\frac{k\nu_{\text{M}}}{U}\left(c_{0}-c_{\text{sat}}\right)-\pi r^{2}\frac{k}{Q}=\frac{U_{0}}{U}\left[1-{\left(\frac{r}{R}\right)}^{2}\right], \end{array}$$

where $U_{0}=k\nu_{\text{M}}\left(c_{0}-c_{\text{sat}}\right)$ is the growth rate at the apex of a stalagmite (r=0) with a horizontal slope (cos θ=1). The ratio $U_{0}/U$ defines a Damköhler number

[9] $$\begin{array}{c} \mathrm{Da}\;=\frac{U_{0}}{U}=\frac{\pi R^{2}k}{Q}, \end{array}$$

where the oversaturation ($c_{0}-c_{\text{sat}}$) enters indirectly, through the radius (Eq. **7**).

The explicit shape of the stalagmite can be obtained by integrating the tangent slope,

[10] $$\begin{array}{c} \frac{\mathrm{dz}}{\mathrm{dr}}=-\tan\theta =-\tan\;\arccos\mathrm{Da}\;\left(1-\frac{r^{2}}{R^{2}}\right), \end{array}$$

which gives rise to a family of shapes with different Damköhler numbers and propagation velocities. The shape function z(r) is an elliptic integral (*SI Appendix*):

[11] $$\begin{array}{cc} \frac{z(r)}{R} & =C-\sqrt{\frac{1}{\mathrm{Da}\;\left(1-\mathrm{Da}\;\right)}}\left[{\mathrm{Da}\;}^{-1}\Pi \left(1+{\mathrm{Da}\;}^{-1};\phi |m\right)\right.\\ & \left.+\left(1-\mathrm{Da}\;\right)E\left(\phi |m\right)-F\left(\phi |m\right)\right], \end{array}$$

where C is a constant that can be fixed to match z(0) to the apex of the stalagmite. The functions F(ϕ|m), E(ϕ|m), and Π(n; ϕ|m) are incomplete elliptic integrals of the first, second, and third kind, with amplitude and parameter

[12] $$\begin{array}{c} \phi =\;\arcsin\sqrt{\frac{\mathrm{Da}\;}{1+\mathrm{Da}\;}}\frac{r}{R},\;\;\;\;m=-\frac{1+\mathrm{Da}\;}{1-\mathrm{Da}\;}. \end{array}$$

**Fig. 3.**
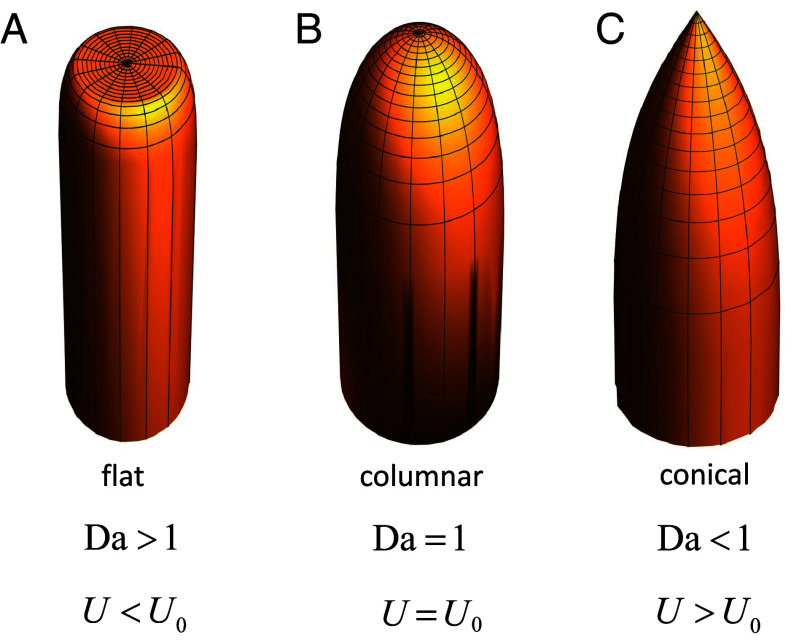
Flat-top (*A*), columnar (*B*), and conical stalagmite (*C*) for $U/U_{0}=1/2,1$, and 2, respectively.

The key results are Eqs. **10** and **11**, which characterize the shapes of invariant stalagmites as a function of a single parameter, the Damköhler number. Numerical simulations (11) found only a single (columnar) shape, growing with an upward velocity $U=U_{0}$, but our theory includes three qualitatively distinct shapes, which propagate with different velocities $U=U_{0}/\mathrm{Da}\;$ (Fig. 3): flat top (Da>1), columnar (Da=1), and conical (Da<1). Stalagmites with shapes similar to these ideal forms can frequently be observed in caves (Fig. 1); we examine each case in more detail in the subsequent sections.

The problem of determining which member of a family of possible solutions will be realized under specific physical conditions is also encountered in solidification and viscous infiltration (16, 27–29). In these systems, shape selection is only possible after incorporating additional physics into the model. We will argue that the key factors in stalagmite shape selection are the distribution and degassing of the dripping water in the vicinity of the stalagmite tip.

## 2. Results

### 2.1. Flat Top Stalagmites: *U* < *U*_0_.

When Da>1, the tangent slope from Eq. **10** is only defined (cos θ≤1) outside a critical radius $R_{c}$):

[13] $$\begin{array}{c} R_{c}=R\sqrt{1-{\mathrm{Da}\;}^{-1}}. \end{array}$$

However, the solution for z(r) given by Eq. **11** can be extended to the interior region by supplementing it with a flat cap $z\left(r\right)=z_{\text{apex}}$ when $r<R_{c}$. Both the shape function and its tangent are then continuous at $r=R_{c}$, and we obtain stalagmites with flat tops (Fig. 3*A*) instead of hemispherical ones (Fig. 3*B*). Interestingly, such forms are quite frequently observed in nature (Fig. 1 *F* and *J*), particularly when the distance between the cave ceiling and the top of the stalagmite is relatively large in comparison with its width.

A rationale for flat caps may be found by considering the falling distance of the droplets wetting the stalagmite surface. The flat tops shown in Fig. 1*F* are located near the cave floor at least 10 m from the ceiling. A droplet falling onto a solid surface from an appreciable height spreads into a thin lamella that covers a significantly wider area than the droplet cross-section (30). Moreover, individual droplets do not necessarily fall in straight lines, due to interactions with the wake; oscillations in the trajectories of falling spheres were noted by both da Vinci and Newton (31, 32). More recently, the spatial distribution of falling droplets inside caves was quantified by Parmentier et al. (33). Their measurements indicate that, for stalagmites far below the ceiling, it is unrealistic to assume that each droplet lands squarely on the apex. Instead, the wetted area is a combination of the distribution of impact points and the impact-induced spreading of the individual droplets. This hypothesis is supported by observations of short flat-top stalagmites in many caves, for example Fig. 1 *F* and *J*.

To account for distributed wetting, we assume that the water flux can be described as a spatially uniform dripping over a circle of radius $R_{c}$, with a rate P (volume per unit area per unit time). In contrast to the point source, the outflow Q is no longer constant. Inside the radius of the wetted area ($r<R_{c}$), $Q\left(r\right)=\pi r^{2}P$, while outside $Q=\pi R_{c}^{2}P$ is constant. The transport equation, including the source of calcium ions from the dripping water, now leads to a constant concentration in the region $r<R_{c}$ (*SI Appendix*),

[14] $$\begin{array}{c} c=c_{0}-\frac{U}{P\nu_{M}}. \end{array}$$

Outside the source region ($r>R_{c}$) the concentration is again given by Eq. **6** with $Q=\pi R_{c}^{2}P$ (*SI Appendix*, Eq. **S27**).

Constant concentration implies that the slope (tanθ) is also constant, as a condition for invariant propagation. Imposing the condition for a flat top stalagmite, $\theta (r<R_{c})=0$, we can obtain the growth velocity from Eq. **5**,

[15] $$\begin{array}{c} U=\frac{k\nu_{M}\left(c_{0}-c_{\text{sat}}\right)}{1\;+\;k/P}=\frac{U_{0}}{1\;+\;k/P}, \end{array}$$

or, from Eq. **9**, Da=1+k/P. The relative size of the stalagmite $R/R_{c}$ (Eq. **13**) and the relative propagation velocity $U/U_{0}$ (Eq. **15**) are uniquely determined by the dripping rate P. The absolute values of these properties can be determined from the radius of the wetted area ($R_{c}$) and the degree of oversaturation $c_{0}-c_{\text{sat}}$.

The dripping rate depends on the volumetric flow impinging on the stalagmite, Q, and the radius of the wetted area $R_{c}$. The flow rate is usually estimated from the size of the droplets and the time between drips, while $R_{c}$ is a complicated function of Q and the fall distance. However, numerical values can be obtained from empirical formulae derived from experimental observations of splashing droplets (33). Beyond the dripping radius the flat-top merges with the general shape from Eq. **11**, with a continuous tangent $\theta (R_{c})=0$. The concentration field c(r) from Eq. **6** is also continuous at $R_{c}$, matching the constant concentration from Eq. **14** (*SI Appendix*).

### 2.2. Columnar Stalagmites: *U* = *U*_0_.

**Fig. 4.**
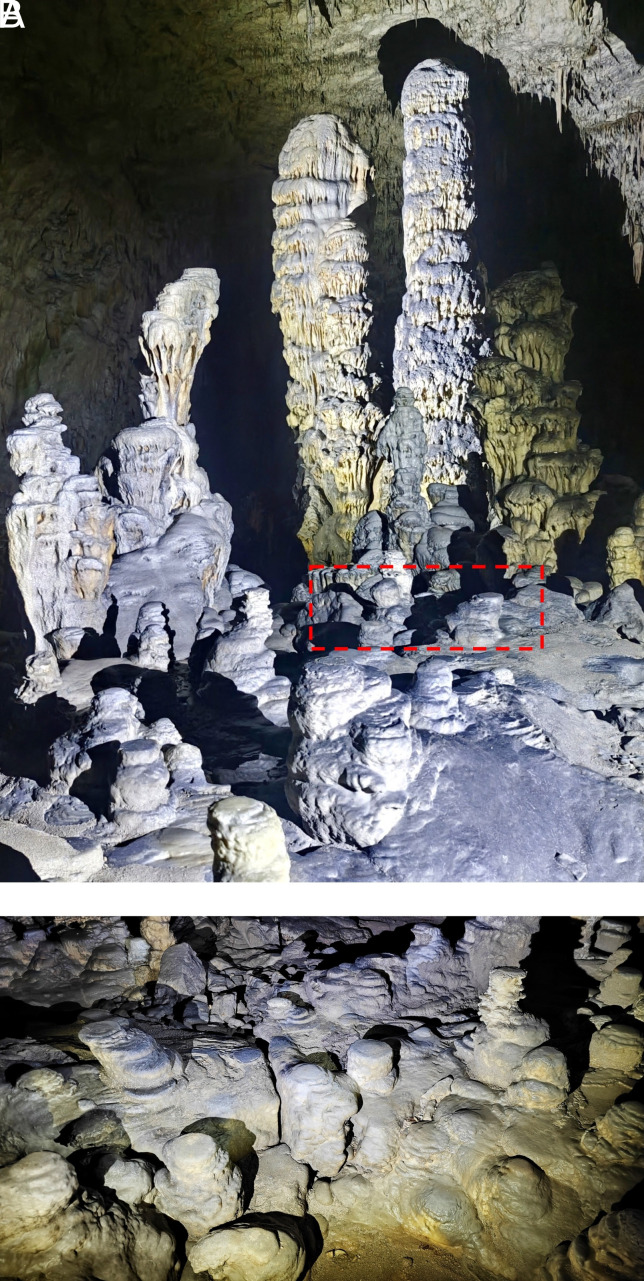
Stalagmites in Postojna Cave (Slovenia). (*A*) Overview of a portion of the cave, showing taller columnar forms with roughly hemispherical caps and low-lying flat-top shapes. (*B*) A close-up photo in the neighborhood of the dashed area in *A*, showing a large number of flat-top stalagmites. Photos by Matej Lipar.

If the spread of the droplets is small in comparison to the radius of the stalagmite $R_{c}\ll R$, the flat top shape approaches the columnar one, with the flat region reduced to a point (Fig. 3*B*). Examples of the effect of fall height can be found in Postojna Cave (Fig. 4). Here, most of the short stalagmites are flat tops (Fig. 4*B*), whereas the taller ones have rounded tops (Fig. 4*A*).

The top of a columnar stalagmite advances with a velocity $U=k\nu_{M}\left(c_{0}-c_{\text{sat}}\right)=U_{0}$. The mass balance from Eq. **7** then requires the stalagmite to adopt a radius $R=\sqrt{Q/\pi k}$. Thus a columnar stalagmite can adapt to different values of Q and k, by adjusting its radius. This is exactly what happens in the numerical simulations of Romanov et al. (11), where the condition $U=U_{0}$ is imposed.

Columnar stalagmites are pillar-like shapes, with a roughly hemispherical cap (Fig. 1*B*, *C*, *E*, *H*, and *L*). They are the prevalent shape in caves, occurring whenever the radius of the wetted area is small in comparison to the stalagmite size: $R_{c}\ll R$ and Da→1 (Eq. **13**). Their shape is a limiting case of (Eq. **11**), which can be expressed in terms of elementary functions (*SI Appendix*):

[16] $$\begin{array}{c} \frac{z(r)}{R}=C+x+\frac{1}{2}\ln\left(\frac{x-1}{x+1}\right), \end{array}$$

where $x=\sqrt{2-{\left(r/R\right)}^{2}}$. Numerical simulations of stalagmite evolution (11) imposed the condition $U=U_{0}$ at the apex of the stalagmite, finding the same final (columnar) shape from a variety of initial conditions and matching well with our analytic solution. From Eq. **8** it can be seen that this boundary condition corresponds to the case Da=1 and θ(0)=0.

### 2.3. Conical Stalagmites: $U>U_{0}$.

Conical stalagmites grow more quickly than columnar shapes with the same degree of oversaturation. They have a positive slope at the origin, cos θ(0)=Da (Eq. **10**), meaning a pointed tip (Fig. 3*C*). In the small Damköhler limit, cos θ≪1 and Eq. **10** simplifies to

[17] $$\begin{array}{c} \frac{\mathrm{dz}}{\mathrm{dr}}=-\frac{1}{\mathrm{Da}\;(1-r^{2}/R^{2})}. \end{array}$$

Eq. **17** can be integrated directly, leading to a particularly simple result,

[18] $$\begin{array}{c} \frac{z(r)}{R}=C-{\mathrm{Da}\;}^{-1}\mathrm{arctanh}\;\frac{r}{R}. \end{array}$$

**Fig. 5.**
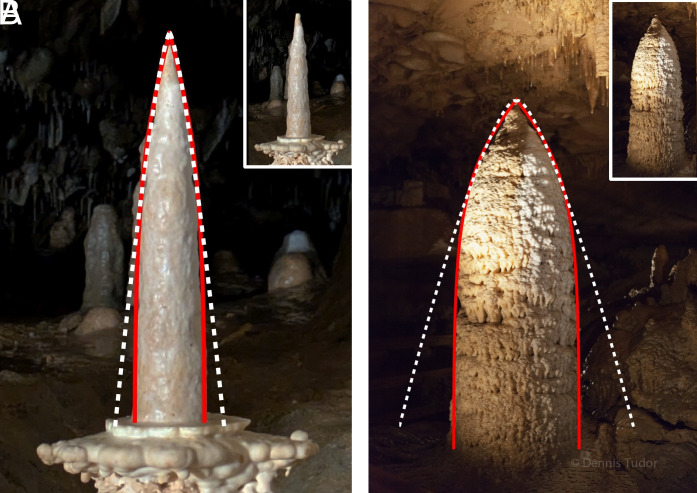
(*A*) “Candlestick” stalagmite in Sloupsko-šošůvské Caves, Czechia, shown together with a fit to the arctanh function (Eq. **18**) (Da = 0.23; red solid line), and a fit to the stalactite solution of ref. 34 (white dashed line). (*B*) A large conical stalagmite from Cub Run Cave, Kentucky, USA, shown together with a fit to the full stalagmite solution (Eq. **11**) (Da = 0.59; red solid line), and a fit to the stalactite solution (white dashed line). *Insets*: Original photographs of the stalagmites. Photo credits: (*A*) Piotr Szymczak, (*B*) Dennis Tudor (reproduced with permission).

For example, the shape of the stalagmite in Fig. 5*A* is well described by the hyperbolic arctangent (red line) from Eq. **18**. The Damköhler number (Da=0.23) and the radius are the only parameters in the fit. On the scale of the figure, the solution is indistinguishable from the exact solution (Eq. **11**).

The Damköhler number of the flat top shape is selected by the radius of the wetting, while the columnar stalagmite is a limiting case when the wetted radius is small. However, a selection mechanism for conical stalagmites is less obvious, but a hint might be found from similarities with stalactites. These are another speleothem with pointed tips, but growing from the roof of the cave rather than the floor. In fact, stalagmites are frequently found growing underneath stalactites, so the same water drips onto both. Nevertheless, stalactites are slender and elongated, much like icicles, whereas stalagmites are considerably bulkier (Fig. 1). Their shapes are not mirror images of each other, which suggests stalactite and stalagmite growth are controlled by different precipitation mechanisms. Here, we attempt to rationalize the different speleothem shapes by following the evolution of a water packet as it falls from the cave ceiling. To do this, we will integrate observations of speleothem growth with the kinetics of calcite precipitation, and with theoretical predictions of the shapes of steadily growing stalactites and stalagmites.

Stalactite growth has been linked to the production of carbonic acid during precipitation, which is followed by dehydration as the rate limiting step (34),

[19] $$\begin{array}{c} {\text{H}}_{2}{\text{CO}}_{3}\to {\text{CO}}_{2}+{\text{H}}_{2}\text{O}. \end{array}$$

In this model, the precipitation rate is proportional to the thickness of the fluid film h (Fig. 2),

[20] $$\begin{array}{c} J(h)=-\alpha h, \end{array}$$

where α is the net rate of conversion of carbonic acid (and its dissociation products $HCO_{3}^{-}$ and $CO_{3}^{2-}$) to aqueous CO_2_ (35). The film thickness is related to the volumetric flow rate and the slope of the surface (Eq. **1**). The linear dependence on h arises because calcite precipitation (proportional to surface area) is assumed to be linked mole-to-mole with the production of carbonic acid, which must be equilibrated with the cave atmosphere by a bulk conversion reaction (proportional to volume). By assuming invariant growth with constant α, Short et al. derived a functional form for stalactites, which for large radii asymptotically approaches $z∼r^{4/3}$. However, it is clear that shapes derived from Eq. **20** do not match the observed stalagmite shapes shown in Fig. 1. Even conical stalagmites do not follow a power law, but look more like the elliptic integral predicted by a surface-limited reaction rate (Eq. **11**). In Fig. 5, analytic solutions from Eq. **11** (shown by the red lines) were fitted to the outlines of conical stalagmites. These solutions fit the observed shapes almost perfectly, while the inverted stalactite derived from Eq. **20** does not capture the basal region of the conical stalagmites (white lines in Fig. 5).

We can perhaps understand the different shapes of stalactites and stalagmites in terms of the carbon content of the precipitating solution. Water entering the cave can have an effective *p*CO_2_ up to 0.1 bar, because of the absorption of biologically sourced carbon as it percolates through the soil (36). Aqueous calcium ions are in equilibrium with this more acidic solution (pH 6.5–7) at considerably higher concentrations (up to 4 mM) than solutions in equilibrium with the cave atmosphere (pH > 8), where the calcium solubility is almost an order of magnitude smaller (0.5 mM). Water flowing down the outside of the stalactite loses CO_2_ to the cave atmosphere by a combination of chemical conversion and transport of dissolved CO_2_. Calcite is deposited on the sides of stalactites, while the water accumulates in droplets at the bottom. The droplets hang for 1 to 300 s (37) before falling to the cave floor or sometimes onto the apex of a stalagmite. We hypothesize that the hanging droplet and the subsequent splashing as it impacts the stalagmite marks the transition point between stalactite-forming kinetics (high *p*CO_2_) and stalagmite-forming kinetics (low *p*CO_2_).

This hypothesis is supported by the existence of two reaction pathways for calcite precipitation (38, 39):

[21] $$\begin{array}{cc} {\left(\text{Ca}-{\text{HCO}}_{3}\right)}^{+}+{\text{HCO}}_{3}^{-}[\text{s}] & \to {\text{CaCO}}_{3}+{\text{H}}_{2}{\text{CO}}_{3}, \end{array}$$

[22] $$\begin{array}{cc} {\left(\text{Ca}-{\text{HCO}}_{3}\right)}^{+}+{\text{OH}}^{-}[\text{s}] & \to {\text{CaCO}}_{3}+{\text{H}}_{2}\text{O}. \end{array}$$

The competition between these pathways is determined by the populations of surface-bound anions ($HCO_{3}^{-}\left[s\right]$ and $OH^{-}\left[s\right]$) in the water layer adjacent to the calcite surface. The first reaction path dominates precipitation when the aqueous carbon concentration is high (stalactite formation), and in thin films the overall rate is limited by the slow conversion of carbonic acid (15, 34). However, as the dissolved carbon comes to equilibrium with the cave atmosphere the pH increases, and the second reaction takes over (39). Since it does not produce carbonic acid, H2CO3 conversion is no longer limiting. The different shapes of stalactites and stalagmites can then be understood in terms of a transition between the different precipitation kinetics: Stalactites precipitate under conditions where the rate is controlled by the conversion of H_2_CO_3_ to CO_2_, while stalagmites precipitate under a surface-limited reaction (Eq. **3**).

Most stalagmites are either flat-tops (distributed source) or columnar (point source), suggesting that stalagmites usually precipitate under conditions where the solution is close to equilibrium with the cave atmosphere. However, in cases where the flow rate is large or when droplets arrive directly from the ceiling, water impacting the apex of the stalagmite may not be fully equilibrated, so the kinetics may again be limited by CO_2_ conversion. Then the initial section of the stalagmite would resemble a mirror reflection of the stalactite. An example of a stalactite–stalagmite pair is the “Romeo and Juliet” speleothem shown in Fig. 1*I*. The top portion of the stalagmite is an approximate mirror image of the stalactite shape, but lower down it adopts the bulkier form more typical of a conical stalagmite. The transition point between the conversion-controlled shape (inverted stalactite) and the surface reaction controlled regime corresponds to the location where the liquid flowing down the stalagmite has spent sufficient time $t_{\text{eq}}$ on the stalagmite surface to equilibrate with the cave atmosphere:

[23] $$\begin{array}{c} t_{\text{eq}}\approx \frac{\delta c_{\text{sat}}}{\alpha }. \end{array}$$

Here, $\delta c_{\text{sat}}$ is the difference between the calcium ion saturation concentration at the top of the stalagmite and the saturation concentration when CO_2_ has equilibrated with the cave air. At this point, the slope of the inverted stalactite $∼r^{1/3}$ (34) and the slope of the conical stalagmite (Eq. **10**) should match, giving a selection rule for the conical Damköhler number.

Fig. 5 provides a striking confirmation of this hypothesis. First, the inverted stalactite solution from Eq. **20** fits the observed conical shapes in the region of the tip, while missing the steepening of the tangent slope when r→R. Second, the extent of the tip region, where the shape can be described by an inverted stalactite, increases as Da is reduced; it is larger for the elongated “Candlestick” with Da=0.23 (Fig. 5*A*) than the blunter shape with Da=0.59 (Fig. 5*B*).

### 2.4. Isotope shifts.

Stalagmites serve as important archives of past climates, offering insights into the Earth’s environmental history. These include variations in shape (5, 10, 11), which can be correlated with variations in rainfall (40), and shifts in isotope abundance due to fractionation during precipitation and degassing (40–42). Their utility in climate studies stems from their unique formation process and the geological information they encapsulate from many millenia. Stalagmites grow in layers, much like tree rings, with each layer representing a snapshot of the environmental conditions at the time of its deposition, thus integrating a record of the isotopic and chemical composition of the environment above the cave. By cross-referencing stalagmite data with other paleoclimate records, such as ice cores and tree rings, a more comprehensive and nuanced picture of Earth’s climatic history can be inferred. However, the interpretation of isotope shifts are complicated by a number of uncertainties, including the kinetic effects of calcium deposition and CO_2_ release on the fractionation rates (43–46).

**Fig. 6.**
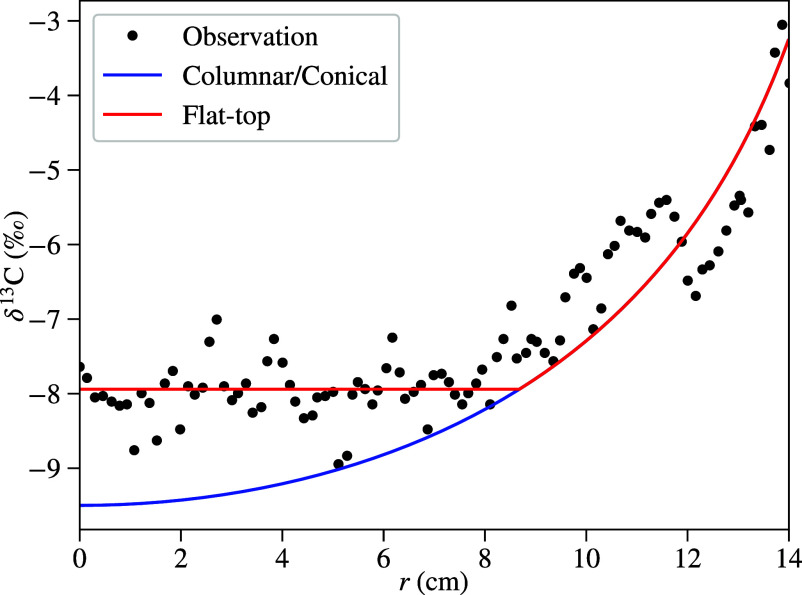
Comparison of predicted and observed isotope shifts. The solid lines show the predicted isotope shifts for flat-top (red) and columnar/conical (blue) stalagmites; the black symbols are experimental measurements from a flat-top stalagmite recovered from Ceremosnia cave (Serbia) (49).

Numerical simulations are increasingly being used to help interpret isotope measurements (7, 46–48), but these studies have so far been applied only to columnar shapes. A key insight from our theoretical analysis is that the morphology of the stalagmite can affect not just the distribution of isotope shifts, but also the shift along the stalagmite centerline. Predicted isotope profiles from our model (*Materials and Methods*) are shown in Fig. 6 for a typical set of cave conditions (48). The parabolic profile (blue line) is characteristic of columnar and conical stalagmites (48), but a distributed water source leads to a qualitatively different prediction. Here, the constant concentration in the flat cap leads to a constant $\delta^{13}\text{C}$ shift until the outer radius of the wetted region ($r=R_{c}$) is reached, after which it follows the shifts for columnar and conical shapes.

Fig. 6 includes experimental measurements of isotope shifts in a large flat-top stalagmite. There is a region (r<9 cm) with a constant $\delta^{13}\text{C}$, followed by an increasing shift in the region beyond the spread of the droplets. The observed shifts can be described semiquantitatively by our calculated concentration for a flat-top stalagmite (red line in Fig. 6). On the other hand, a columnar or conical stalagmite would show an isotope shift that follows the blue curve, because of the parabolic concentration profile. Thus, morphological information (flat-top vs. columnar or conical) can be an important and hitherto unrecognized factor in the correct interpretation of isotope measurements. The analytic results obtained in this work provide a means to separate environmental from morphological influences on $\delta^{13}\text{C}$ and $\delta^{18}O$ shifts. By recording radial profiles in different vertical slices, transitions from flat-top to columnar (or conical) shapes could be detected.

## 3. Discussion

The analytic solutions derived in this work provide a comprehensive framework for understanding the shapes of steadily growing stalagmites, unifying previous numerical and empirical observations into a coherent theory. We showed that Franke’s model of stalagmite growth admits an analytic solution and that this solution describes a family of shapes parameterized by a single quantity–the Damköhler number, Da. Previously, only the case Da=1 was discussed in the literature (and only numerically, without analytic results), corresponding to columnar stalagmites. As it turns out, however, the same formalism also describes flat-top stalagmites for Da<1 and conical forms for Da>1.

**Fig. 7.**
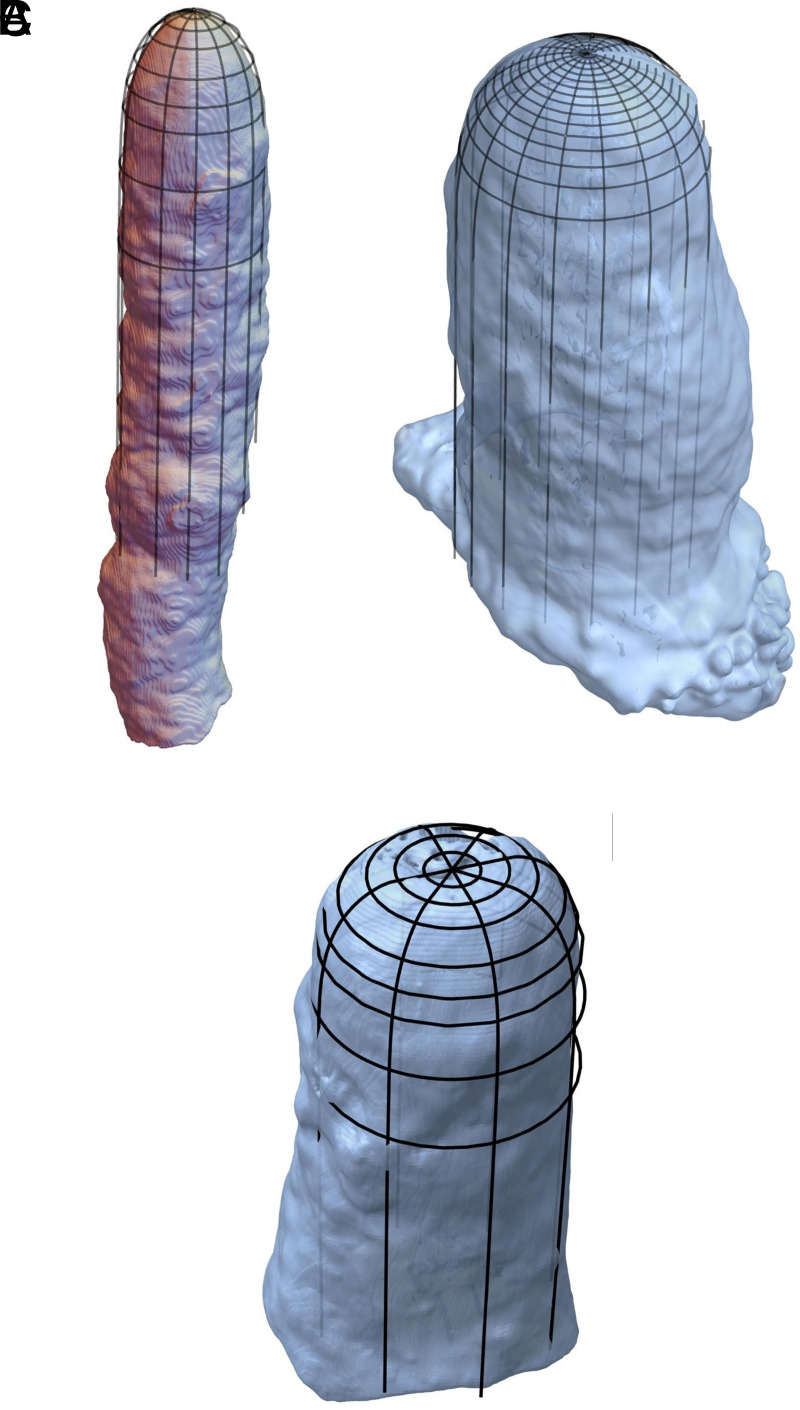
Tomographs of stalagmites from Postojna Cave (Slovenia) together with the superimposed shape from Eq. **10**. (*A*) Columnar stalagmite with radius R≈7 cm. (*B*) Columnar stalagmite with radius R≈12 cm. (*C*) Flat-top stalagmite with radii $R_{c}\approx 2.9$ cm and R≈6.5 cm.

All of these shapes are observed in caves, as evidenced not only by photographs but also by the direct comparison of shape profiles. We performed X-ray tomography on several small stalagmites from Postojna Cave, which had been removed for paleoclimatic studies, and then overlaid the analytic profiles predicted by Eq. **11** onto the tomography data, as shown in Fig. 7. The two first examples (*A* and *B*) correspond to columnar stalagmites (Da=1), where the only fitting parameter is the radius, R. The third example (*C*) shows a flat-top stalagmite, for which we fit both the base radius and the radius of the flat top, $R_{c}$. All of the predicted shapes match the real specimens remarkably well, especially given that the analytic profiles assume idealized conditions (such as a steady dripping rate) that are not necessarily realized in natural cave environments.

As with most cases of invariant growth in pattern-forming systems, we encountered a shape selection problem: The solutions of our equations describe a continuous family of shapes and additional physics must be introduced to determine which shape is realized under specific environmental conditions. For flat-top stalagmites, we were able to link selection to the dripping rate and the area over which droplets are splashed: These two quantities uniquely determine the shape of the growing stalagmite. We have hypothesized that shape selection in conical stalagmites is connected to a shift in chemical kinetics; from a regime limited by the conversion of carbonic acid to ${\mathrm{CO}}_{2}$, to one limited by the surface kinetics of the precipitation reaction.

The model suggests that transitions between different shapes are continuous, controlled by the value of Da. However Da is a consequence of selection rules, which depend on the extent of degassing, the radius of wetting, and the volumetric flow rate. If the solution dripping onto a stalagmite is more or less in equilibrium with the cave atmosphere, as is generally the case, then whenever the fall distance is small enough that the radius of the wetting $R_{c}\ll R$, a columnar shape will emerge. This may explain why columnar stalagmites are so prevalent; some of them even have small flat tops indicating a small but finite wetting radius.

Observations of stalagmite size and shape can be used to infer flow rates. First, the Damköhler number is estimated by fitting Eq. **11** to the stalagmite shape, as in Figs. 5 and 7. Second, the stalagmite radius is used in conjunction with Eq. **9** to determine the flow rate. For example, the stalagmites shown in Fig. 7 have flow rates that are estimated to be (taking $k=1.3\times 10^{-5}$ cm/s): A. 0.002 cm^3^/s, B. 0.0045 cm^3^/s, and C. 0.001 cm^3^/s. These correspond to dripping times (assuming a droplet volume of 0.1 cm^3^) of 50 s, 22 s, and 100 s.

Finally, our model reveals that the shape of a stalagmite influences its isotope profile. For instance, flat-top stalagmites exhibit a constant $\delta^{13}\text{C}$ shift in their central region, while columnar and conical forms display parabolic profiles. These differences should be accounted for when interpreting isotopic records, as they reflect morphological rather than environmental variations in calcium ion concentration. By incorporating our analytic solutions, researchers can refine climate reconstructions and extract more accurate information from speleothem archives.

Several limitations of this study need to be acknowledged. Our analytic solutions describe stalagmites that grow under perfectly steady, axisymmetric, laminar flow, yet several cave-specific factors fall outside this idealization. When drip rates become very low the water no longer forms a continuous film: Individual droplets strike the apex, spread as transient rivulets, and precipitate until they reach saturation after only a few centimeters of travel. That finite travel distance imposes a lower limit on the basal diameter—the “smallest-stalagmite” puzzle first posed by Curl (12)—which any continuum thin-film model necessarily misses. In a related problem of icicle growth, laboratory imaging shows that such rivulets wet the surface only patchily, leaving large dry zones and altering the ripple growth (50); an analogous incomplete wetting on stalagmites could likewise modify their growth dynamics and trigger shape instabilities in ways the uniform-film theory cannot capture. Additionally, in real caves the boundary conditions themselves drift; slow seasonal or secular drifts would drive the system through a sequence of quasi-steady shapes, with the profile at any moment reflecting the entire past evolution of Da(t), not just its value at that instant. Cylindrical symmetry is another idealization: Lateral air currents and inclined drip trajectories introduce three-dimensional asymmetries that only numerical models or laboratory analogues can quantify.

In summary, this study provides a unified theory for the shapes of ideal stalagmites, grounded in the principles of reactive transport. The derived solutions not only explain the diversity of observed forms but also enhance the utility of stalagmites as paleoclimate proxies.

## 4. Materials and Methods

### 4.1. Isotope Data.

Carbon isotope fractionation can be expressed in terms of the shift in the isotope ratio $R={[}^{13}\text{C}]/{[}^{12}C]$ from the reference $R_{\text{VPDB}}$ expressed in parts per mille (‰). By following the kinetics of precipitation and degassing from a water packet flowing over the stalagmite, isotope shifts can be predicted (48):

[24] $$\begin{array}{c} \delta^{13}\text{C}=\left[\alpha_{1}\frac{R_{{\mathrm{HCO}}_{3}^{-}}^{0}}{R_{\text{VPDB}}}{\left(\frac{c}{c_{0}}\right)}^{\alpha_{1}+\alpha_{2}-1}-1\right]\times 1000. \end{array}$$

Here, we have made use of the fact that the carbon content of the solution is over 95% ${\mathrm{HCO}}_{3}^{-}$, together with the electroneutrality condition, which for pH>6 connects the bicarbonate and calcium concentrations ${\mathrm{HCO}}_{3}^{-}=2Ca^{2+}$. The fractionation coefficients $\alpha_{1}$ and $\alpha_{2}$ relate to the two chemical reactions involving carbon: the deposition of CaCO_3_ and the outgassing of CO_2_ (48). Consistent with the cave conditions in Fig. 6 (49), we assume equilibrium fractionation of the isotopes in determining $\alpha_{1}$ and $\alpha_{2}$. We took these values and the initial calcium concentration (2.6 Mol/m^3^) from Romanov et al. (48).

To fit the experimental measurements, we assumed an average flat top radius of 8.7 cm. The size of the flat cap was not specified but the overall dimensions were 10 to 18 cm (49). We took the $\delta^{13}\text{C}$ shift in the dripping water to be −9.5‰ relative the Vienna standard $R_{\text{VPDB}}$, which is then used to determine the initial isotope ratio $R_{{\mathrm{HCO}}_{3}^{-}}^{0}$. These parameters can only be regarded as representative, because we have no specific information on their values. However the qualitative features of the flat-top isotope profiles—the reduced shift at the centerline compared to the columnar case, and the flat profile in the wetted region—are independent of the choice of parameters.

### 4.2. Tomography.

Stalagmites for computed tomography (CT) scans were collected in the Pisani Rov Passage of the Postojna Cave, Slovenia. They were all found lying on the ground beneath an approximately 10 m high cave ceiling, broken by the movement of collapsed rubble and clay. The CT was performed with a Siemens Dual Source CT Somatom Force at the Institute of Radiology, University Medical Centre (UMC) Ljubljana, Slovenia. The X-ray beam was oriented perpendicular to the object and, based on the beam spectrum and attenuation coefficient of the scanned materials, the data were processed in the detector system.

A dual energy protocol was used to gain better spectral separation of the composition. First, we performed posterior-anterior (PA) and lateral (LAT) scans with a tube voltage of 120 kV and a current of 20 mA. We used those images to plan the start and end of the dual energy scan. For exposure parameters, a detector collimation of 128×0.6 mm was used. A tube voltage of 100 kV was applied, with currents of 190 mA in the first X-ray tube, and 380 mA in the second X-ray tube. A tin filter was used to filter out low-energy X-rays. For the final images, a slice thickness of 1.0 mm with 0.7 mm increments was used, with a tube voltage of Sn 150 kV.

## Supplementary Material

Appendix 01 (PDF)

## Data Availability

All study data are included in the article and/or *SI Appendix*.
