# Supplementary material for: Shapes of ideal stalagmites
Source: Proc Natl Acad Sci U S A. 2025 Oct 16;122(42):e2513263122. doi: 10.1073/pnas.2513263122 (PMC12557760; doi:10.1073/pnas.2513263122)
Supplement: Supplementary file 1 — Appendix 01 (PDF) [file pnas.2513263122.sapp.pdf]

Supporting Information for

Shapes of ideal stalagmites

Piotr Szymczak, Anthony J.C. Ladd, Matej Lipar, Dean Pekarovič

piotrek@fuw.edu.pl, tladd@che.ufl.edu

This PDF file includes:

Supporting text
SI References

Supporting Information Text

The Supporting Information contains notes on evaluating the elliptic integral in Eq. 11, a derivation of the axisymmetric transport balance Eq. 4, and a more detailed derivation of the concentration field in Eq. 14.

Evaluating the elliptic integrals. From Eq. 10, the tangent slope of a steadily growing stalagmite is:

$$\frac{dz}{dr} = -\tan\theta = -\frac{\sqrt{u^2 - (1-(r/R)^2)^2}}{1-(r/R)^2}, \tag{S1}$$

where $u = U/U_0 = \mathrm{Da}^{-1}$. When $u \geq 1$ $\tan\theta$ is real for all $r \in [0, R]$, but when $u < 1$ $\tan\theta$ is only real in the range $R\sqrt{1-u} \leq r < R$, which corresponds to the exterior region of a flat top stalagmite. The interior region is created by a distribution of droplets, but outside of the source radius, $\tan\theta$ is the same as for the point water source. Here we consider both the full-range ($0 \leq r < R$) solution when $u \geq 1$ and the exterior solution ($R_c \leq r < R$) when $u < 1$.

The integral of [S1] is an elliptic integral (1), which can be reduced to a linear combination of the canonical Legendre forms:

$$F(\phi|m) = \int_0^{\sin\phi} \frac{1}{\sqrt{(1-mt^2)(1-t^2)}}dt, \tag{S2}$$

$$E(\phi|m) = \int_0^{\sin\phi} \frac{1-mt^2}{\sqrt{(1-mt^2)(1-t^2)}}dt, \tag{S3}$$

$$\Pi(n;\phi|m) = \int_0^{\sin\phi} \frac{1}{(1-nt^2)\sqrt{(1-mt^2)(1-t^2)}}dt, \tag{S4}$$

where the elliptic integrals have been expressed in terms of the amplitude ϕ and modulus m. There are general formulas for the reduction of elliptic integrals (1), but these can lead to unnecessarily complex expressions for $z(r)$. Instead we follow the reduction algorithm, making use of the simplifications allowed by the functional form of $\tan\theta$.

Defining the polynomials P and Q in terms of $\hat{r} = r/R$,

$$P(\hat{r}) = u^2 - (1-\hat{r}^2)^2, \quad Q(\hat{r}) = 1 - \hat{r}^2, \tag{S5}$$

the shape function can be found from the integral

$$\frac{z(r)}{R} = -\int \frac{P}{Q\sqrt{P}}d\hat{r} + C, \tag{S6}$$

where C is to be chosen so that $z(0)$ coincides with the apex of the stalagmite z_{apex}. The ratio P/Q is reducible to a proper rational function

$$\frac{P(\hat{r})}{Q(\hat{r})} = \frac{u^2}{1-\hat{r}^2} - 1 + \hat{r}^2, \tag{S7}$$

and P can be factored into quadratic terms,

$$P(\hat{r}) = \left(u - 1 + \hat{r}^2\right)\left(u + 1 - \hat{r}^2\right) = (u^2-1)\left(1 + \frac{\hat{r}^2}{u-1}\right)\left(1 - \frac{\hat{r}^2}{u+1}\right). \tag{S8}$$

We will choose the definition of the amplitude so that the angle remains real and in the range $[0, \pi/2]$:

$$t = \sin\phi = \hat{r}/\sqrt{u+1}, \quad m = -\frac{u+1}{u-1}, \quad \sqrt{P(t)} = \sqrt{(u^2-1)}\sqrt{(1-mt^2)(1-t^2)}. \tag{S9}$$

However the parameter m is not necessarily bounded from above by $m = 1$ and the integrand becomes complex when $mt^2 > 1$.

To connect the integral in [S6] to the canonical elliptic integrals [S2]–[S4], we define the auxiliary function

$$\zeta(r) = z_{\mathrm{apex}} - z(r), \tag{S10}$$

which is the vertical distance from the apex of the stalagmite to the point $z(r)$. From the substitutions in [S9], we have

$$\frac{\zeta(r)}{R} = \sqrt{1/(u-1)}\int_0^{\sin\phi}\left(\frac{u^2}{1-(u+1)t^2} + (u-1)(1-mt^2) - u\right)\frac{dt}{\sqrt{(1-mt^2)(1-t^2)}}, \tag{S11}$$

where $\phi = \arcsin(r/\sqrt{u+1})$. In some cases ($u < 1$, and $r < \sqrt{1-u}$) $\zeta(r)$ is a complex function.

The terms in parentheses include the three Legendre forms of elliptic integrals:

$$\frac{\zeta(r)}{R} = \sqrt{1/(u-1)}\left[u^2\Pi\left(u+1;\phi|m\right) + (u-1)E\left(\phi|m\right) - uF\left(\phi|m\right)\right], \tag{S12}$$

where the square root should be evaluated as written (rather than $1/\sqrt{u-1}$) to obtain the correct sign when $u < 1$.

$\underline{u > 1}$. For $u > 1$, ζ is real and the evolution of the invariant conical shape is

$$z(r,t) = z_{\text{apex}}(t) - \zeta(r/R), \tag{S13}$$

where $z_{\text{apex}}(t)$ is the time-dependent position of the apex of the stalagmite. The elliptic integral $\Pi(u+1;\phi,m)$ contains a logarithmic divergence near $r = R$, which is the origin of the vertical outer boundary.

$\underline{u = 1}$. We cannot write the solution for $u = 1$ in terms of elliptic integrals, since the modulus $m \to \infty$. However, the polynomial P simplifies to $P(\hat{r}) = \hat{r}^2(2-\hat{r}^2)$, suggesting the substitution $x = \sqrt{2-\hat{r}^2}$. The resulting integrand is a rational function of x and can be integrated to give

$$\frac{\zeta(x)}{R} = -x - \frac{1}{2}\log\frac{x-1}{x+1}. \tag{S14}$$

The invariant shape evolves as,

$$z(r,t) = z_{\text{apex}}(t) - \zeta(\sqrt{2-\hat{r}^2}) + \zeta(\sqrt{2}). \tag{S15}$$

This function also has a logarithmic divergence near $r = 1$.

$\underline{u < 1}$. The tangent slope becomes imaginary when $r < R\sqrt{1-u}$, so solutions of the form of [S11] are not applicable. Outside of the source region the equations are the same as for a point source, and the exterior shape $(r > R_c)$ is then given by

$$z(r,t) = z_{\text{apex}}(t) - \zeta(r/R) + \zeta(R_c/R). \tag{S16}$$

Since $\Im(\zeta)$ is constant in the region $R_c <= r < R$, $z(r,t)$ is real and continuous at $r = R_c$.

Derivation of Eq. 4: axisymmetric transport balance. We consider a thin, well-mixed film flowing axisymmetrically over a surface $z(r)$ with local tangent angle $\theta(r)$. Under the fast cross-film diffusion $(kh/D \ll 1)$, the calcium concentration is uniform across the film thickness and depends only on r: $c = c(r)$. The depth-integrated volumetric flow around a circumference of radius r is a constant Q, while the depth-integrated flux of Ca^{2+} is $Q\,c(r)$.

Consider an annular control volume between radii r and $r + \mathrm{d}r$. The net decrease of solute flux through the annulus equals the precipitation sink on the rock surface within the annulus:

$$\partial_r(Qc)\,\mathrm{d}r \;=\; -\,J(c)\,\mathrm{d}A_{\text{surf}}, \tag{S17}$$

where $J(c)$ is the precipitation rate per unit surface area ($\mathrm{mol\,m^{-2}\,s^{-1}}$), and $\mathrm{d}A_{\text{surf}}$ is the wetted rock area. Geometry gives $\mathrm{d}A_{\text{surf}} = 2\pi r \mathrm{d}s = 2\pi r \mathrm{d}r/\cos\theta$, because the surface arc length $\mathrm{d}s = \mathrm{d}r/\cos\theta$ for a slope θ.

With linear kinetics $J(c) = k\,(c - c_{\text{sat}})$, and dividing [S17] by $2\pi r dr$ yields Eq. 4 in the main text,

$$\frac{1}{2\pi r}\,\partial_r(Qc) \;=\; -\,\frac{k\,(c-c_{\text{sat}})}{\cos\theta}, \tag{S18}$$

The boundary condition is $c(0) = c_0$.

Concentration field under a distributed source. The distribution of droplets coupled with their impact-based spreading (2) suggests that the water source can be described as a uniform dripping over a circle of radius R_c, with the dripping rate $P = Q/(\pi R_c^2)$. A mass balance within an annular region $2\pi r dr$ $(r < R_c)$ is similar to [S18],

$$\frac{1}{2\pi r}\partial_r\,(Qc) = -\frac{k(c-c_{sat})}{\cos\theta} + Pc_0, \tag{S19}$$

but with two important differences. First, the volumetric flow is now r-dependent,

$$Q(r) = \pi r^2 P. \tag{S20}$$

Second, there is an extra source term Pc_0 arising from the dripping of oversaturated solution bringing additional calcium ions.

For an invariantly growing stalagmite, the first term on the RHS can be replaced by $-U/\nu_{\mathrm{M}}$,

$$\frac{1}{2\pi r}\partial_r\left(\pi r^2 Pc\right) = -\frac{U}{\nu_{\mathrm{M}}} + Pc_0. \tag{S21}$$

Equation (S21) can be readily integrated to show that the concentration under a uniformly distributed source is constant:

$$c = c_0 - \frac{U}{P\nu_{\mathrm{M}}}. \tag{S22}$$

The integration constant from [S19] must vanish to prevent the concentration diverging at the origin.

Constant concentration implies that the tangent slope (θ) will also be constant, as a condition for invariant propagation:

$$\cos\theta(r) = \frac{k\nu_{\mathrm{M}}}{U}(c - c_{\text{sat}}) = \frac{k\nu_{\mathrm{M}}}{U}(c_0 - c_{\text{sat}}) - \frac{k}{P} \tag{S23}$$

For a flat surface ($\theta(r) = 0$), the propagation velocity is determined by the ratio of the reaction rate constant to the dripping rate P,

$$U = \frac{k\nu_{\mathrm{M}}(c_0 - c_{\mathrm{sat}})}{1 + k/P} = \frac{U_0}{1 + k/P}. \tag{S24}$$

The outer radius of the stalagmite is determined by conservation of calcium ions in the growing stalagmite,

$$\pi R^2 U/v_M = Q(R_c)(c_0 - c_{\mathrm{sat}}), \tag{S25}$$

and the stalagmite radius is then related to the radius of the wetting

$$R = R_c\sqrt{1 + P/k}. \tag{S26}$$

The constant concentration field in the flat region [S22] connects continuously to the outer region (Eq. 6),

$$c(r > R_c) = c_0 - \frac{\pi r^2 U}{Q(R_c)\nu_{\mathrm{M}}} = c_0 - (c_0 - c_{\mathrm{sat}})\frac{r^2}{R^2}, \tag{S27}$$

where the last equality follows from [S25].

References

1. M Abramowitz, IA Stegun, *Handbook of Mathematical Functions.* (Dover, New York), (1972).
2. J Parmentier, et al., A drop does not fall in a straight line: a rationale for the width of stalagmites. *Proc. Royal Soc. A* **475**, 20190556 (2019).
